# A Population Survey of Dietary Attitudes towards Gluten

**DOI:** 10.3390/nu11061276

**Published:** 2019-06-05

**Authors:** Iain D Croall, Nick Trott, Anupam Rej, Imran Aziz, David J O’Brien, Harvey A George, Mohammed Y Hossain, Lauren J S Marks, Jessica I Richardson, Rebecca Rigby, Marios Hadjivassiliou, Nigel Hoggard, David S Sanders

**Affiliations:** 1 University of Sheffield, Academic Unit of Radiology, Royal Hallamshire Hospital, Sheffield S10 2JF, UK; i.croall@sheffield.ac.uk (I.D.C.); n.hoggard@sheffield.ac.uk (N.H.); 2 Academic Unit of Gastroenterology, Royal Hallamshire Hospital, Sheffield Teaching Hospitals NHS Foundation Trust, Sheffield S10 2JF, UK; Nick.Trott@sth.nhs.uk (N.T.); a.rej@sheffield.ac.uk (A.R.); Imran.Aziz@sth.nhs.uk (I.A.); dobrien2@sheffield.ac.uk (D.J.O.); hageorge1@sheffield.ac.uk (H.A.G.); myhossain1@sheffield.ac.uk (M.Y.H.); laurenmarks76@outlook.com (L.J.S.M.); jirichardson1@sheffield.ac.uk (J.I.R.); rrigby1@sheffield.ac.uk (R.R.); 3 Department of Neurology, Royal Hallamshire Hospital, Sheffield Teaching Hospitals NHS Foundation Trust, Sheffield, S10 2JF, UK; m.hadjivassiliou@sheffield.ac.uk

**Keywords:** gluten sensitivity, population survey, people who avoid gluten, lifestylers, coeliac disease

## Abstract

It is unclear how the prevalence of people who believe the gluten-free diet (GFD) to be generally healthy (“Lifestylers”) is impacting the overall rates of self-reported gluten sensitivity (GS). We repeated a population survey from 2012 in order to examine how attitudes towards GS have changed over time. Our survey (*N* = 1004) was administered in Sheffield (UK) in 2015, replicating the 2012 experiment. The questionnaire included a food frequency survey and assessed self-reported GS as well as associated variables (prevalence, current diet, pre-existing conditions, etc.). The overall rates of key variables and chi-squared analysis in comparison to the previous survey were as follows: self-reported GS was 32.8% (previously 12.9%, *p* < 0.001), pre-existing coeliac disease (CD) was 1.2% (previously 0.8%, *p* = 0.370), following a GFD was 3.7% (previously 3.7%, *p* = 0.997). Self-reported GS was positively associated with some pre-existing conditions, including anxiety, depression, chronic fatigue, headaches, and other food allergies/intolerances (including irritable bowel syndrome (IBS); chi-squared analyses, all *p* < 0.001). Over a 3-year period, the fraction of people who self-reported GS increased by over 250%. Despite this, arguably more meaningful indications of underlying physiological GS remained comparable. This research suggests that the public perception of gluten is causing a marked increase in the number of people who erroneously believe they are sensitive to it.

## 1. Introduction

Although studies repeatedly indicate that the prevalence of coeliac disease (CD) is approximately 0.5%–1% in the United Kingdom (UK) and American populations [1,2,3], the elimination and restriction of gluten from diets has increased dramatically in recent years. The total value of the global gluten-free food industry has grown from $1.7bn in 2011 to $3.5bn in 2016 and is forecast to reach $4.7bn in 2020 [4]. Non-coeliac gluten sensitivity (NCGS) [5] describes a physiological sensitivity to gluten in the absence of CD or wheat allergy (WA), and this population undoubtedly contributes to the popularity of the gluten-free diet (GFD) outside of CD patients. Although the precise nature of NCGS is the subject of some debate, notable studies have shown findings which support gluten as being a causative agent in this syndrome [6,7,8,9]. However, celebrity and athletic endorsements of the GFD have also cultivated an image that it is healthy for all people [10]. This is not supported by the scientific literature, which continues to assert that gluten is safe to eat for individuals without any forms of gluten sensitivity (GS) [11]. Encouraged by this perception of healthy eating, many people now eat gluten-free food as a lifestyle choice. These are known as “Lifestylers”, “clean eaters”, “free from”, or sometimes “people who avoid gluten”.

Scientific research assessing the prevalence and effects of this belief is lacking, although indications may be inferred from other sources. International Google Trends data concerning the search term “gluten free healthy” (accessed on 1 May 2019) show that the search has steadily increased in popularity from 2009, before peaking in 2014. It has remained very popular with an interest level in the third quartile (when compared to the first quartile prior to 2009). This implies approximately five times as much interest if the middle values of each quartile are compared (i.e., 12.5 vs. 62.5). Lifestylers also appear to account for a substantial proportion of those engaged with a GFD. American market research conducted in 2013 [12] and 2014 [13] found that 44% of people who buy gluten-free food do it for reasons other than any kind of GS, and that 65% of people believed that a GFD was generally healthier. A qualitative study performed in the UK assessing the attitudes of people with NCGS also uncovered some responses which indicated a perception of gluten as being generally unhealthy [14], which suggests that Lifestyler beliefs are also now directly influencing the self-reporting of GS.

Additional dilution of the NCGS population likely occurs due to the overlap of symptoms with other food intolerances, such as irritable bowel syndrome (IBS) [15]. Indeed, it is thought that this is one reason why some NCGS studies show negative results [16]. It would be expected that the high level of Lifestyler interest in a GFD would further increase the possibility of people over-attributing such similar symptoms to it, and in others may encourage a “nocebo” effect upon commencing a GFD. As the self-reporting of symptoms due to gluten is the central diagnostic feature for NCGS, it is important that the clinical and public perception of gluten remains balanced. The surge in gluten-free popularity has also led to an opposing belief that it is a “fad” diet [17,18]. This can affect people with CD/NCGS, who find that their condition is taken less seriously in contexts such as eating out at a restaurant [19].

In 2012 we conducted a UK population-based questionnaire which found that while 12.9% of respondents reported GS, those who were on a GFD only accounted for 3.7% [20]. As clinically diagnosed NCGS often causes severe symptoms and thus provides strong motivation for patients to adopt a GFD, such a disparity may be indicative of a Lifestyler demographic with a less notable and/or specific presentation. We undertook a population-based survey to re-characterise the number of people who self-report gluten sensitivity since that survey, and then investigate associated phenomena such as amounts of dietary gluten consumed and the co-occurrence of other conditions.

## 2. Materials and Methods

### 2.1. Participants

Participants were recruited outside a large shopping centre in Sheffield, UK between September and October 2015. This was the same as the main recruitment location from the previous survey conducted by the group in 2012 [20] so that changes over the intervening 3 years could be comparably assessed. The only criteria were that the subjects were aged 16 or older. There were 1004 people recruited for the study that completed the questionnaire.

The population survey was supported by personal research funds of Prof. Sanders. The project was discussed with Sheffield Teaching Hospitals Foundation Trust and the regional Research Ethics Committee prior to commencement, and as the study was a public survey conducted entirely outside of healthcare services, it was determined that formal ethical approval was not required. Regardless, the project fell within the remit of a “blanket” ethical approval granted by the Yorkshire & Humber (Sheffield) Research Ethics Committee, for research into CD, potential CD, and gluten sensitivity. The project was otherwise conducted in accordance with the principles outlined in the Declaration of Helsinki.

### 2.2. Methods

The questionnaire was a modified version of the one previously administered in 2012 [20] (which has also been implemented in other settings [21,22]). It was first used to assess dietary gluten via a food frequency survey (adapted from others which have been validated in children [23,24]). It was then used to collect demographic information and characterise whether the respondent had any of a number of pre-existing medical conditions. Finally, the questionnaire was used to identify if the subject self-reported a GS, and then interrogated associated variables, including specific symptoms (and their course), if the subject has ever been assessed by a healthcare professional for GS (and if they received a CD or WA diagnosis), and if they were on, or had ever tried a GFD. The questionnaire is available in the Supplementary Material as Item S1.

### 2.3. Data Analysis

All analysis was conducted in SPSS version 23. Key variables were summarised so that a descriptive comparison could be made to the accompanying study that was conducted 3 years previously. X^2^ tests were used to separately analyse if the fraction of respondents who self-reported GS, adhered to a GFD, or had CD were significantly different between these studies. The overall fraction of self-reported GS/GFD adherence included those with diagnosed gluten-related disorders in order for these rates to be wholly comparable between surveys.

Statistical analyses were then conducted to more closely examine the features of people who self-reported a GS *without* having been diagnosed CD or WA (i.e., those who appear to have a NCGS). The X^2^ analyses examined the relationship between NCGS and the occurrence of pre-existing medical conditions. After a visual inspection of the histogram to confirm normality, the mean daily consumption of gluten (calculated from the food frequency questionnaire) was compared by a *t*-test between the NCGS and non-GS groups.

## 3. Results

### 3.1. Overall Population Characteristics

The 1004 subjects that were recruited into the questionnaire had a mean age of 36.6 years (range = 16–86, SD = 17.2), and 59.2% were female. The overall mean amount of gluten consumed daily was 13.2 g (SD = 9.2). The rate of people reporting pre-existing gastrointestinal disorders was as follows: stomach or bowel cancer = 0% (*N* = 0), gastroesophageal reflux disease (“reflux”) = 6.4% (*N* = 64), irritable bowel disease = 0.3% (*N* = 3). IBS was reported as a pre-existing condition without any of these diseases (or CD; characterised later) in 4.4% of respondents (*N* = 44) and was reported with an overall rate of 5.2%.

### 3.2. Self-Reported Gluten Sensitivity

**Figure 1:**
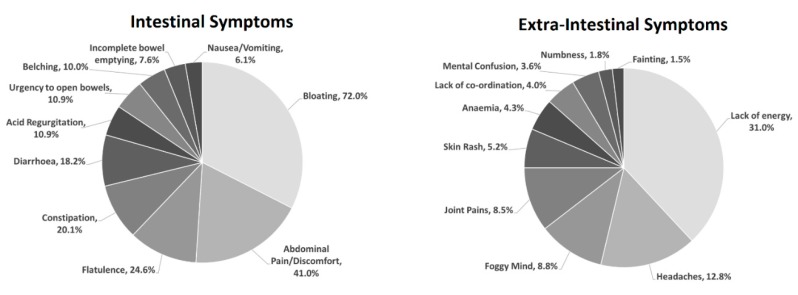
Pie charts showing the distribution of intestinal (**left**) and extra-intestinal (**right**) symptoms amongst respondents who self-reported gluten sensitivity.

The rate of self-reported GS was 32.8% (*N* = 329). This sub-group had a mean age of 37.7 (SD = 16.0) and was 66.4% female. The most reported symptom was bloating (72.0%), followed by abdominal pain and discomfort (41.0%), lack of energy (31.0%), flatulence (24.6%), and constipation (20.1%). Diarrhoea, headaches, acid reflux, urgency to open bowels, and belching were all reported at rates between 10%–20% (inclusive). Foggy mind, joint pains, feelings of incomplete bowel emptying, nausea/vomiting, and skin rashes were reported between 5%–10%, while anaemia, co-ordination problems, mental confusion, numbness/tingling, and fainting were all reported at <5%. This is visualised in Figure 1.

Of the overall self-reported GS subgroup (i.e., including those with diagnosed gluten-related disorders), 25.5% had previously tried a GFD, of whom 75.0% said this helped with their symptoms (the remaining 25.0% either answered that the diet did not help or that they were not sure). The total proportion of people who self-reported GS, and who were presently on a GFD at the time of the questionnaire was 11.2% (*N* = 37, meaning the overall rate of people on a GFD within the whole population was 3.7%). In people who self-reported a GS, 14.0% said they had a reaction every time they ate gluten, while 19.1% had seen a healthcare professional at some point about their symptoms, leading to 3.6% of them to receive a CD diagnosis (*N* = 12) and 1.5% of them to receive a WA diagnosis (*N* = 5). The overall rate of CD within the whole population was therefore 1.2%, while the overall rate of self-reported GS without CD or WA (i.e., NCGS) was 31.1%. All respondents who reported CD indicated they had been diagnosed via formal clinical testing that included coeliac blood tests in all 12 cases, and also an endoscopy in 11 out of 12 cases (also reported: skin prick allergy test in *N* = 3, colonoscopy in *N* = 2, biopsy in *N* = 1, and lactose intolerance test in *N* = 1).

Table 1 gives an overview of the key variables contained in both the 2012 and 2015 questionnaires, for a descriptive comparison [20]. To summarise, we had previously reported an overall rate of self-reported GS (including diagnosed gluten-related disorders) at 12.9% (presently 32.8%), with a clinical diagnosis of CD at 0.8% (presently 1.2%), and the total number of people on a GFD at 3.7% (presently also 3.7%). The chi-squared analysis showed the difference in GS reporting rates to be highly significant (*p* < 0.001), while GFD (*p* = 0.997) and CD (*p* = 0.370) were non-significant.

Further analyses focused on comparisons between those with NCGS (i.e., self-reported GS without CD or WA) and respondents who did not report any form of GS. Within the current questionnaire, daily gluten intake of the NCGS group (mean = 11.3 g, SD = 8.2) was significantly lower than in people without self-reported GS (mean = 14.4 g, SD = 9.4, independent *t*-test *p* < 0.001). X^2^ analyses showed that the NCGS group more often reported pre-existing disorders of anxiety (NCGS = 22.4%, non-GS = 8.0%, *p* < 0.001), depression (NCGS = 14.7%, non-GS = 4.4%, *p* < 0.001), chronic fatigue (NCGS = 3.5%, non-GS = 0.6%, *p* < 0.001), myalgic encephalomyelitis (NCGS = 10.3%, non-GS = 3.4%, *p* < 0.001), chronic headaches (NCGS = 9.0%, non-GS = 2.7%, *p* < 0.001), egg allergy (NCGS = 3.2%, non-GS = 0.3%, *p* < 0.001), dairy intolerance (NCGS = 5.1%, non-GS = 0.7%, *p* < 0.001), acid reflux (NCGS = 11.2%, non-GS = 4.1%, *p* < 0.001), and IBS (NCGS = 9.0%, non-GS = 3.3%, *p* < 0.001). These findings all survived the Bonferroni correction. Provisionally significant results of the same direction (i.e., *p* < 0.05, where NCGS respondents more often reported the condition), but which did not survive this correction, were also reported for thyroid disease and fibromyalgia. Note that for comparison with the previous survey (which also investigated the occurrence of pre-existing conditions, but in the overall self-reporting GS group), these findings remain significant if including those with CD/WA (see Table 1 for comparisons).

## 4. Discussion

This population survey indicates that in 2015, nearly a third of the general UK population perceived that they had symptoms related to the ingestion of gluten. This is in contrast to a comparable survey conducted in 2012, which found a much lower prevalence, suggesting that there was a dramatic increase in the number of people in the UK who believe they have GS, despite there being no underlying change in the number of individuals on a GFD.

Patients who self-report symptoms related to gluten must have CD excluded, although the rate of self-reported GS presented here is far higher than any estimate of CD prevalence, even when taking undiagnosed cases into account [1]. Physiological GS may also exist as NCGS. NCGS is often the subject of debate, although notably a meta-analysis of randomised placebo-controlled trials investigating symptomatic response to gluten did indicate an overall positive effect when included studies were limited to those which followed consensus criteria for the definition and investigation of the syndrome [6] (the “Salerno” criteria [5]). Additionally, other literature has demonstrated physiological changes in NCGS populations when compared to controls or CD patients, indicating that gluten may cause an activation of the innate immune system [9]. The sum of this research suggests that sensitivity to gluten does exist outside of CD and WA. However, as effective clinical testing for NCGS remains unavailable, the self-reporting of gluten-related symptoms is the most pertinent diagnostic criteria. This emphasises the importance of a balance in the public’s understanding of the reactions that can be caused by gluten.

NCGS is thought to affect between 0.5%–13% of the population [25], meaning the overall rate of self-reported GS found in the present study is remarkably high even when taking this population into account. There are a number of reasons why an overestimation of self-reported GS may be occurring. The overlap between NCGS and IBS is close, and it has been proposed that many individuals experiencing IBS [15] or other similar intolerances [16] may erroneously confuse their condition as being gluten-driven. However, the rate of people with underlying food sensitivities is unlikely to have changed between 2012–2015, so other factors must be considered. Scientific studies specifically investigating the impact of “Lifestyler” beliefs within the NCGS population are lacking, but these attitudes likely hold a strong influence. One qualitative study assessing the beliefs of an NCGS group detected some Lifestyler-type responses, including statements such as “Grains and sugar are slowly killing us all!!” [14]. A rise in such beliefs may additionally encourage the self-misdiagnosis of IBS (by making gluten a more prominent consideration), and also lead to “nocebo” effects after eating gluten.

Our 2012 survey was conducted using a comparable population-based questionnaire where we found that 12.8% of people self-reported GS [20]. The contemporary survey’s finding of 32.7% therefore represents a highly significant increase of over 250%. On the other hand, in the present study the number of people who had CD (1.1%) or were on a GFD (3.8%) were not different from this previous experiment (0.8% and 3.7%, respectively). Overall IBS prevalence (without other gastrointestinal conditions) was also comparable, with rates of 4.4% and 6% in the present/previous study (for reference these rates are similar to the lower end of the UK IBS prevalence estimates—6.1% [26]). Therefore, despite a marked rise in self-reported GS, behaviours and diagnoses which would meaningfully support that this increase has a physiological basis did not change. Of relevance to this, it should also be noted that this disparity between the rate of people who self-report GS and the fraction of those who adhere to a GFD is far greater here than has been found in similar studies in Mexico [27] (overall GS; 11.9%, GFD; 3.7%) and Colombia [28] (overall GS; 7.9%, GFD; 5.9%). Our finding that 25% of GS subjects who had tried a GFD did not report that it helped further shows that for many people who label themselves as gluten-sensitive, the perceived connection between gluten and the symptoms it causes does not need to be clear.

Overall our results suggest that the reasons for the increase in self-reported GS are likely driven by factors other than clear physiological GS, supporting the hypothesis that the misplaced belief that gluten is generally unhealthy has influenced this. Our findings scientifically demonstrate that the number of people who self-report GS is rising, and suggest that the increase in Lifestyler beliefs as evidenced by Google Trends between 2009–2014 (detailed in the Introduction) is leading to this.

A number of results from the questionnaire did however draw some links between NCGS (i.e., self-reported GS without diagnosed CD or WA) and clinical outcomes. People with NCGS overall consumed less daily gluten, implying some level of restriction, and more often reported a range of pre-existing conditions including depression, anxiety, headaches, fatigue, reflux, and IBS. These show parity with symptoms and conditions associated with CD [29,30]. Notably however, a study which investigated Lifestylers specifically reported an opposing finding by showing depression to occur at a lower rate in this group [31]. It is likely that our results stem from those within the NCGS group who do have clinical conditions. These may either be people with correctly identified NCGS, or people with other related disorders such as IBS who are mistaking their symptoms for GS. There is no reason to suspect that the prevalence of people with these physiological susceptibilities has increased since the 2012 questionnaire, meaning that the number of people within the total self-reported GS population who do have disorders is probably becoming more diluted.

There are some limitations to this study. Some potentially valuable details were not collected in the survey. Further work would benefit from directly assessing information, such as whether respondents understand what gluten is, if they believe it is *generally* unhealthy to eat, or their given reason for believing they are GS (aside from attributing some symptoms to it; this would be particularly valuable in the sub-population who do not claim to benefit from a GFD).

In conclusion, this survey provides scientific evidence that the gluten-free “fad” has now led to a sharp increase in the number of people who believe they have GS. However, current clinical opinion supports that gluten is fine for the people without a demonstrable sensitivity to it. As the GFD has been suggested as an overall sub-optimal diet [32], if anything there is a possible need for dissuading the notion that gluten is generally unhealthy. Further, as the prevalence of the diet has also encouraged scepticism of it in a number of people [17], there is a risk that such continued interest will cause patients with physiological GS (CD, NCGS, etc.) to be treated less compassionately in pertinent settings such as restaurants [15]. This study therefore provides some important impetus in grounding the public and clinical perspectives on the topic of gluten sensitivity.

## Figures and Tables

**Table 1:** Comparison of the key variables between the current study (conducted in 2015) and the 2012 questionnaire it replicates. The *p*-values indicate X^2^ analyses to compare the rate of self-reported gluten sensitivity, coeliac disease (CD), and an adherence to a gluten-free diet (GFD).

Variable	As Reported in 2012 (Overall *N* = 1002)	As Reported in The Current Study (Measured in 2015, Overall *N* = 1004)	Chi-Squared *p* Value (Where Conducted)
Prevalence of self-reported gluten sensitivity	12.9% (*N* = 129)	32.8% (*N* = 329)	*p* < 0.001
Mean age (years) of people with self-reported gluten sensitivity	39.5 ± 17.7	37.7 ± 16.0	-
% Female within people with self-reported gluten sensitivity	79%	66%	-
Overall prevalence of coeliac disease diagnosis	0.8% (*N* = 8)	1.2% (*N* = 12)	*p* = 0.370
Overall prevalence of people adhering to a GFD	3.7% (*N* = 37)	3.7% (*N* = 37)	*p* = 0.997
Pre-existing conditions which gluten-sensitive individuals are more likely to have	Anxiety, Depression, Chronic fatigue syndrome, Food allergies/intolerances, IBS (rates not reported)	Anxiety (22.4%), Depression (14.7%), Chronic fatigue syndrome (3.5%), Myalgic encephalomyelitis (10.3%), Chronic headaches (9.0%), Acid reflux (11.2%), Food allergies/intolerances/IBS (3.2%–9.0%)	-

## Supplementary Materials

The following are available online at https://www.mdpi.com/2072-6643/11/6/1276/s1, Item S1: Gluten Food Frequency Questionnaire.
