# Supplementary material for: A Population Survey of Dietary Attitudes towards Gluten
Source: Nutrients. 2019 Jun 5;11(6):1276. doi: 10.3390/nu11061276 (PMC6628309; doi:10.3390/nu11061276)
Supplement: Supplementary file 1 [file nutrients-11-01276-s001.pdf]

Gluten Food Frequency Questionnaire

Food	Portion Size (g)		Frequency of Consumption										Gluten per portion (g)	Total Gluten (g)
			Never	Once per month or less	Once per Fortnight	Number of days per week								
BREADS														
White Sliced	Small	31											2.1	
	Medium	36											2.4	
	Large	44											3	
Wholemeal Sliced	Small	25											1.8	
	Medium	36											2.6	
	Large	44											3.2	
Baguette	2" Slice	40											3.1	
	6" Slice	120											9.2	
	12" Slice	240											18.4	
Roll (white / Wholmeal)	6" Roll	112											8.7	
	Hamburger	60											4.1	
	3" Roll	45											3.3	
Bagel	Average	90											7.6	
Crumpet	Average	40											1.9	
Croissant	Average	60											3.9	
Pitta Bread	Average	75											5.5	
	Large	95											7	
Pikletes	Average	25											1.4	
Malt Loaf	Average Slice	35											2.3	
Chapattis	Average	55											4.0	
Naan	Average	140											9.0	
Brioche	Individual	45											2.9	
	Slice	65											4.2	
BISCUITS														
Rich Tea	Average	7											0.4	
Digestive	Average	15											0.8	
Jaffa Cakes	Average	13											0.5	
Caramel Wafer	Average	27											0.8	
Choc Chip	Average	7											0.3	
Penguin	Average	25											1.1	
Kit Kat 2 fingers	Average	22											1.0	
Garibaldi	Average	10											0.4	
Ginger Nuts	Average	10											0.4	
Iced Ring	Average	12											0.5	
Oatcake	Average	13											1.0	
Wagon Wheel	Average	38											1.4	
Flapjack	Average	70											3.3	
	Small	30											1.4	
Water Biscuits	Average	3.4											0.3	

Food	Portion Size (g)		Frequency of Consumption										Gluten (g)	Total Gluten (g)
			Never	Once per month or less	Once per Fortnight	Number of days per week								
SAVOURY SNACKS														
Crisp-bread	Average	10											0.7	
Ritz Crackers	Average Portion	25											1.5	
Nik Naks	Average Portion	25											1.0	
Pringles	Average Portion	30											0.9	
Mini Cheddars	Average Bag	25											2.1	
Twiglets	Average Portion	25											2.5	
Pretzels	Average Portion	30											2.4	
Hula Hoops	Average Bag	24											0.6	
Wholegrain Bites	Average Portion	30											1.8	
Onion Rings	Average Portion	30											1.5	
CAKES														
Almond Slice	Average	35											1.7	
Apple Tart	Average Slice	88											3.7	
Bakewell Tart	Average Slice	120											6.6	
Battenberg	Average Slice	32											1.5	
Black Forest Gateau	Average Portion	90											2.7	
Chelsea Bun	Average	78											4.9	
Cherry Bakewell	Average	46											1.4	
Cupcake	Average	60											1.9	
Chocolate Éclair	Average	90											4.0	
Current Bun	Average	60											3.6	
Sponge pudding	Average Slice	110											3.0	
Doughnut	Average	60											2.7	
Eccles Cake	Average	45											1.4	
Fruit Cake	Average	60											2.4	
Fairy Cake	Average	30											1.2	
Hot Cross Bun	Average	50											1.3	

Mince Pie	Individual	55											1.8	
Scones	Average	50											3.0	
Tea Cake	Toasted	55											3.9	
Vanilla Slice	Average	113											4.0	

**Pasta**														
Penne / Fusilli / Macaroni / Spaghetti / Lasagne	Small	50											5	
	Medium	75											7.5	
	Large	100											10	
**Breakfast Cereals**														
Bran Flakes	Small	30											2.6	
	Medium	60											5.3	
	Large	75											6.6	
All Bran	Small	30											3.4	
	Medium	60											6.7	
	Large	75											8.4	
Fruit and Fibre	Small	30											1.9	
	Medium	60											3.8	
	Large	75											4.8	
Malted Wheat’s	Small	30											2.5	
	Medium	60											5.0	
	Large	75											6.2	
Muesli	Small	30											2.6	
	Medium	60											5.3	
	Large	75											6.6	
Multigrain Hoops	Small	30											1.9	
	Medium	60											3.8	
	Large	75											4.8	
Raisin Wheats	Small	30											2.0	
	Medium	60											3.9	
	Large	75											4.9	
Special Flakes	Small	30											2.2	
	Medium	60											4.3	
	Large	75											5.4	
Wheat Biscuits	2 Biscuits	28											2.7	
	3 Biscuits	42											4.0	
	4 Biscuits	56											5.4	
Oats	Small	30											2.4	
	Medium	40											3.2	
	Large	60											4.8	

Medical Questionnaire On Gastrointestinal Symptoms

Dear Sir/Madam

We would be grateful if you would kindly complete this 1-5 minute questionnaire survey as part of a project being undertaken by the Gastroenterology department at the Royal Hallamshire Hospital, Sheffield. There are two parts to this questionnaire and although it asks about your bowel symptoms and past medical history, it is anonymous and the results will remain confidential. You may find parts of this questionnaire repetitive but please try and answer all the appropriate questions tailored for you. Should you have any queries or difficulties completing this survey, please ask our helpful young student doctors!

Thank you for your co-operation and time in completing this questionnaire

Part 1: This asks for basic information about yourself, any abdominal symptoms and your general state of health

Q1) Age _________

Q2) Male ☐ or Female ☐

Q3) Employed ☐, Unemployed ☐, Disabled ☐, Retired ☐

Q4) Single ☐, In a relationship ☐, Divorced ☐, Widowed ☐

Q5) Race : White ☐, Black ☐, Asian ☐, Other ☐ (please state________________)

Q6) Post code ______________________

Q7) Have you suffered with episodes of abdominal pains or discomfort for the last 6 months or more?

Yes ☐ No ☐ - **- if No, please go to Q16**

Q8) If yes, how many days in a month do you approximately experience these abdominal pains or discomfort?

One day a month ☐ Four days a month ☐

Two days a month ☐ 5-10 days a month ☐

Three days a month ☐ More than 10 days a month ☐

Q9) Do you suffer from abdominal bloating (feeling full of gas)? Yes ☐ No ☐

Q10) Do you feel an improvement in your abdominal pains or discomfort after you have emptied your bowels? Yes ☐ No ☐

Q11) Was the start of your abdominal pains or discomfort associated with a change in your bowel frequency? Yes ☐ No ☐

Q12) If yes to Q11, how would you best describe the predominant change in bowel habit?

Diarrhoea ☐ , Constipation ☐ , Alternates between Diarrhoea & Constipation ☐

Q13) Was the start of your abdominal pains or discomfort associated with a change in stool consistency? Yes ☐ No ☐

Q14) If yes to Q13, how best would you describe your stool motions?

Loose, watery or sloppy ☐ Hard, pellet like ☐

Combination of loose at times and hard other times ☐

Q15) Do your abdominal and bowel symptoms get worse with stress? Yes ☐ No ☐

Q16) Are you known to suffer from any of the following? (tick as many that apply)

Anxiety	☐	Chronic headaches	☐
Depression	☐	Nut allergy	☐
Bipolar disorder	☐	Egg allergy	☐
Schizophrenia	☐	Dairy product intolerance	☐
Thyroid disease	☐	Bowel cancer	☐
Young onset diabetes (childhood/early adulthood)	☐	Stomach cancer	☐
Breast Cancer	☐	Heartburn/reflux	☐
Pernicious anaemia (low vitamin B12)	☐	Irritable bowel syndrome	☐
Chronic fatigue	☐	Coeliac disease	☐
Fibromyalgia	☐	Inflammatory bowel disease	☐
ME	☐		

Part 2 – Gluten related symptoms

This part of the questionnaire focuses on whether you develop problems when you eat gluten. Gluten is a product found in wheat, barley or rye. Therefore, it is found in common everyday diets such as cereal, bread, cakes, biscuits, pasta, pizza etc. Should you have any queries or difficulties completing this survey, please ask our helpful young student doctors!

Q1) Do you experience any of the following symptoms which you relate to eating gluten based products? (tick as many that apply)

Bloating (feel full of air) ☐	Headaches ☐
Abdominal Pain ☐	Mental confusion ☐
Abdominal discomfort ☐	Lack of co-ordination ☐
Acid Regurgitation ☐	Nausea/ Vomiting ☐
Diarrhoea ☐	Urgency to open bowels ☐
Constipation ☐	Feeling of incomplete bowel emptying ☐
Numbness/pins & needles ☐	Fainting ☐
Lack of energy ☐	Foggy Mind ☐
Belching ☐	Skin Rash ☐
Flatulence ☐	Joint Pains ☐
Anaemia ☐	

Others (please specify) ____

If you do not suffer from any gluten related symptoms, do not proceed. Thank you

Q2) If yes, how often do you experience symptoms after eating gluten products?

Every time I eat gluten products ☐	Few times a month ☐
On most occasions/days ☐	Few times a year ☐
Few days a week ☐	

Q3) How soon after eating gluten products do you develop symptoms?

Almost immediately (less than one hour) ☐	The next day ☐
1-6 hours later ☐	A few days later ☐
6-24 hrs later ☐	

Q4) How long do your symptoms generally last for?

Minutes ☐ Hours ☐ Days ☐ Weeks ☐ Months ☐

Q5) Which gluten product(s) seems to cause problems? (tick as many that apply)

Bread ☐	Pizza ☐	Cakes ☐
Cereal ☐	Pasta ☐	Biscuits ☐
Porridge ☐	Others (please state) _________________________	

Q6) How long have you had a problem related to gluten?

(state approximate number) _____ months or ______years

Q7) Have you ever seen a healthcare professional due to problems related to gluten? Yes ☐ No ☐

Q8) If yes, please state whom you have seen? (tick as many that apply)

GP ☐ , Hospital doctor ☐, dietician ☐, other ☐(please state) ____________

Q9) If yes, have you undergone any of the following tests to look <u>specifically</u> for a cause as to why you have problems related to gluten? (tick as many that apply)

Coeliac blood test	Yes ☐	No ☐	Not sure ☐
Skin prick allergy test	Yes ☐	No ☐	Not sure ☐
Endoscopy (camera into stomach)	Yes ☐	No ☐	Not sure ☐
You have had no tests at all	Yes ☐		

Other tests (please state) ____

Q10) If yes to Q7, Q8, Q9 have you been given any of the following diagnosis? (please ask the student doctor to explain the different conditions in more detail)

Coeliac disease☐, Wheat allergy ☐, Coeliac disease has been excluded ☐,

No explanation given ☐ , You are not sure ☐, other ☐ _________________________

Q11) Have your ever tried a gluten free diet? Yes ☐ No ☐

Q12) If yes, was it beneficial for your symptoms? Yes ☐ No ☐ Not sure☐

Q13) If yes to Q11, Are you still on a gluten free diet ? Yes ☐ No ☐

Thank You – there are no further questions
